# Late-life suicide: machine learning predictors from a large European longitudinal cohort

**DOI:** 10.3389/fpsyt.2024.1455247

**Published:** 2024-09-17

**Authors:** Nicola Meda, Josephine Zammarrelli, Fabio Sambataro, Diego De Leo

**Affiliations:** ^1^ Department of Neuroscience, University of Padova, Padova, Italy; ^2^ De Leo Fund, Research Division, Padova, Italy; ^3^ Padova University Hospital, Padova, Italy; ^4^ Padova Neuroscience Center, University of Padova, Padova, Italy; ^5^ Italian Psychogeriatric Association, Padova, Italy; ^6^ Australian Institute for Suicide Research and Prevention, Griffith University, Mt Gravatt Campus, Brisbane, QLD, Australia; ^7^ Slovene Centre for Suicide Research, Primorska University, Koper, Slovenia

**Keywords:** suicide, old adults, belongingness, social connection, illness duration

## Abstract

**Background:**

People in late adulthood die by suicide at the highest rate worldwide. However, there are still no tools to help predict the risk of death from suicide in old age. Here, we leveraged the Survey of Health, Ageing, and Retirement in Europe (SHARE) prospective dataset to train and test a machine learning model to identify predictors for suicide in late life.

**Methods:**

Of more than 16,000 deaths recorded, 74 were suicides. We matched 73 individuals who died by suicide with people who died by accident, according to sex (28.8% female in the total sample), age at death (67 ± 16.4 years), suicidal ideation (measured with the EURO-D scale), and the number of chronic illnesses. A random forest algorithm was trained on demographic data, physical health, depression, and cognitive functioning to extract essential variables for predicting death from suicide and then tested on the test set.

**Results:**

The random forest algorithm had an accuracy of 79% (95% CI 0.60-0.92, p = 0.002), a sensitivity of.80, and a specificity of.78. Among the variables contributing to the model performance, the three most important factors were how long the participant was ill before death, the frequency of contact with the next of kin and the number of offspring still alive.

**Conclusions:**

Prospective clinical and social information can predict death from suicide with good accuracy in late adulthood. Most of the variables that surfaced as risk factors can be attributed to the construct of social connectedness, which has been shown to play a decisive role in suicide in late life.

## Introduction

Suicide among people of old age is a serious public health concern. It is well-known that populations around the world are getting older (1), and this trend sets a growing concern about the need to address the issue of suicide risk among middle-aged and older adults.

Globally, from 1990 to 2017, age-standardized suicide death rates decreased by 32.7% (2), and quality of life and access to health care improved (3). However, suicide rates among individuals aged 65 and over are still the highest among men and women in nearly all regions of the world (2, 4–6). In general, suicide rates tend to increase with advancing age: suicide in old age affects 27.45 individuals per 100,000 population in the age group over 70 years and approximately 17 people per 100,000 inhabitants in the 50-69 age group (5). Moreover, suicide rates are underestimated, especially among older people (7). In addition, the unequivocal attribution of a manner of death to suicide can sometimes be challenging due to a possible attempt of the person to disguise the suicidal intent (e.g., as in some single road traffic deaths (8),) or due to the peculiar circumstances of death (e.g., as in house accidents or falls (3, 7)).

Epidemiologists forecast that, in less than 30 years (9), there may be an almost doubling of the older adult population and a growing percentage of single-nuclear families. This prediction leaves researchers and mental health professionals afraid of an increase in loneliness and dependence, which are factors frequently implicated in suicidal behavior (10, 11). The aging process and death from suicide are two psychosocial phenomena linked by a multidimensional and multifactorial nature (12). Old age is generally associated with a decline in physical and mental functions and an increase in chronic and physical diseases that often result in functional limitations and disabilities (13). Changes in social status and loss of social networks and family support are also commonly experienced as individuals age. These are stressors that can affect the quality of life of individuals and increase vulnerability to mental health problems and suicide risk (14–16). Although several risk factors for suicidal behavior have been identified, practical tools to accurately predict which individuals, especially older people, will attempt or die by suicide are substantially lacking (17). A prevention-oriented risk assessment implies identifying longitudinal predictors that may significantly increase the odds of death from suicide and that can be addressed (i.e., mitigated) to decrease the risk of suicide (18, 19).

Important recommendations to improve suicide risk assessment include considering contextual and sociocultural factors of suicidal behavior (20, 21) and possibly leveraging large amounts of data and machine learning techniques to increase the calculation capacity and the possibility of identifying people at risk (22–24). The combination of all available information (data from questionnaires and socio-demographic and clinical factors) could provide better assessment capabilities for preventing suicide (25–28). Noteworthily, given the higher lethality of suicide attempts in late life [lethal to non-lethal suicide attempt ratio is 1:4 in late life and 1:200 in young adults (29)], there is potentially no room for secondary prevention. Thus, effective primary prevention of suicide is the most meaningful outcome to pursue in this population.

Implementing a machine learning-based predictive approach in suicide prevention offers solutions to the challenges of modeling complex, high-dimensional data with non-linear relationships. Overcoming these challenges with common statistical approaches is non-trivial. In particular, random forest models assess the importance of different features in predicting suicide risk while being less prone to overfitting compared to logistic regression.

In this study, we queried the large prospective dataset from the *Survey of Health, Aging, and Retirement in Europe* (SHARE) (30, 31), comprising more than 16,000 deaths from different causes (and 74 deaths from suicide), to develop a machine learning algorithm for suicide prediction in older people. We matched the case and control samples by suicide ideation and then implemented a random forest model, as previously done to predict suicide (32, 33). The expedient of matching by suicidal ideation ensures that the model will not rely on this variable. This choice is crucial for two reasons. The first is statistical, as suicidal ideation is only moderately associated with death by suicide (34); thus, the model could heavily bias its classification based on another suicide-related variable; the second is practical, as suicidal ideation (as well as death thoughts/wishes) can remain undisclosed (35, 36) or be denied (37). We hypothesized that social and physical health-related variables would be the most important variables that the algorithm leveraged to predict suicide (13–16). Thus, we aimed at developing a proof-of-concept algorithm as well as testing the association between constructs of social (dis)connectedness and suicide in older adults.

## Materials and methods

### Dataset curation

The *Survey of Health, Aging, and Retirement in Europe* (SHARE) (30, 31) is a research infrastructure that collects prospective data on physical, mental, social, and economic well-being and independence in activities of daily living of nationally representative samples of people aged 50 or over in Europe. Participants are residents of 28 European nations and Israel. Sampling bias was addressed by SHARE researchers by sampling SHARE participants using probability selection methods. This dataset is coordinated by the Munich Center for the Economics of Aging (MEA) in collaboration with the English Longitudinal Study of Aging (ELSA) and the U.S. Health and Retirement Study (HRS). Since 2005, the research consortium has collected data on the abovementioned variables in eight “waves.” From the second wave onward, data on the cause, manner, circumstances, and antecedents (up to 1 year before) of death were collected if a participant died in the period between the waves. In such a case, the next-of-kin completed an interview as a proxy interviewee. From wave 2 to wave 9 (excluding wave three for reasons explained below), 16,548 deaths were recorded. Study design, sampling, and data resources for SHARE are described in detail elsewhere (30) and can be found online (https://share-eric.eu/).

Data retrieved from the SHARE dataset were managed with RStudio 4.1.2 (38). First, we grouped all interviews after death for each *n*-wave. We matched the information collected therein to the health, demographic, and social factors that the participant, who then died, provided in the *n-1* wave. We chose the interview preceding death as the baseline for the following reason: each participant, unless they dropped out of the study, had completed at least two interviews (one by themselves, the second by the next of kin had death occurred) and, among participants, the number of completed interviews (waves) could vary enough to make a data analysis plan non-trivial.

Since data on participants’ mental health in wave three were not collected as in other waves, we opted not to consider the deaths that occurred in wave four. We thus obtained a dataset that matched before-death information on mental, physical, social, and economic well-being to the cause of death and its circumstances for each wave from 2005 to 2020. The variables considered in the analysis were demographics and household variables (e.g., household size, help received), antecedents to death, behavioral risks (e.g., smoking), cognitive functioning, financial and economic health, physical health (including measurement of grip strength), mental health (e.g., depressive symptoms). See the **Supplementary Material** for the complete list of variables (**Supplementary Table S1**). Given that the baseline measures could have varied from wave to wave, we harmonized the dataset by removing the variables not shared across the waves (all the variables included in the analysis are reported in **Supplementary Appendix Table S1**).

### Statistical analysis

In the seven waves analyzed, 74 deaths by suicide were observed. One of such deaths was later not included in the analysis as the age of death was not collected. We matched the participants who died by suicide one-on-one, by nearest neighbor rule (39), with participants who died in an accident [as in (40, 41)] by gender, age at death, number of chronic physical illnesses, and wish to be dead (present, absent, “do not know” and refuse to report). If data regarding the outcome of interest (i.e., manner of death) or variables on which matching was performed (e.g., suicidal ideation/age at death) were missing, the participant(s) were excluded from further analysis. We used the default and most common matching technique for the nearest neighbor method, propensity score difference (42, 43).

Suicidal ideation was assessed by the interviewer with EURO-D scale item 4: “In the last month, have you felt that you would rather be dead?”. Any mention of suicidal feelings or wishing to be dead was marked as the presence of at least some degree of wish to die. Therefore, active suicidal ideation and passive suicidal ideation were not differentiated in this dataset. The remaining NA values were imputed using median values of the same variable per timepoint (baseline or follow-up) (44). Furthermore, we transformed two variables, the frequency of contact with next-of-kin and the duration of illness, from categorical predictors (e.g., daily contacts = 6, …, weekly contacts = 4) to continuous numeric variables, since we thought that these data would be more informative in a numeric format.

Lastly, the dataset was divided 80/20 into two subsets [train and test (45)]. The larger subset (deaths by suicide = 59; deaths by accident = 58) was used to train a multivariable random forest model in R [(46) see also Methods S1] and thus identify which factors could be leveraged to distinguish death from suicide from death from accident; the remaining subset was used to test the metrics of the model (sensitivity, specificity, overall accuracy, etc.). We opted to employ random forest algorithms because we expected the dataset to contain a large number of potentially useful predictors, with some of them being collinear or interacting with each other in a non-linear fashion. Moreover, random forests deal well with high dimensional data (47). It has also been shown to be effective even with small sample sizes (48). A random forest is an ensemble learning method used for classification and regression tasks in machine learning. In our case, the random forest for classification starts by creating multiple bootstrap samples. For each bootstrap sample, a decision tree is constructed. Instead of considering all features (variables) for splitting at each decision tree node, a random subset of features is chosen. This process makes the trees decorrelated and reduces overfitting.

Once all decision trees have been constructed, predictions are made for each tree. For classification tasks, each tree’s prediction is considered a “vote,” and the class with the most votes becomes the final prediction. Performance metrics to evaluate the performance of a random forest model could be accuracy, sensitivity, and specificity. Data visualization was aided by the *randomForestExplainer* package (49).

## Results

### Population characteristics

A total of 146 individuals were included in this analysis (**Table 1**): half died by suicide, and the other half by accident. Most of the participants lived in Estonia (14.4%), Belgium (10.3%), France (8.9%), Austria (8.2%), Czech Republic (7.5%), and Greece (6.8%), while the rest were from various European countries. Approximately 75% of the deceased were men, and the total sample age of death was 68.05 ± 16.41 years. The next-of-kin who answered the after-death interview was the partner in 40.4% of cases and a son/daughter in 19.2% of cases; notably, a non-relative in approximately 1 out of 4 deceased (a next-of-kin could have also been a neighbor or someone helping in the house). The mean number of children still alive at the participant’s death was 1.90 ± 1.92. Regarding when the deceased had contact with next-of-kin in the last year, in most cases (59.6%), the contact occurred daily. In 1 in 10 cases, the contacts occurred less than once a month or never in the last year.

**Table 1:** Sample characteristics of the overall sample and after stratification for cause of death.

		Overall	Accident	Suicide	*p-value*
**Sample N**		146	73	73	
**Country (%)**	Austria	12 (8.2)	5 (6.8)	7 (9.6)	n.s.
	Belgium	15 (10.3)	4 (5.5)	11 (15.1)	
	Czech Republic	11 (7.5)	4 (5.5)	7 (9.6)	
	Denmark	5 (3.4)	1 (1.4)	4 (5.5)	
	Estonia	21 (14.4)	8 (11.0)	13 (17.8)	
	France	13 (8.9)	4 (5.5)	9 (12.3)	
	Germany	4 (2.7)	3 (4.1)	1 (1.4)	
	Greece	10 (6.8)	10 (13.7)	0 (0.0)	
	Hungary	3 (2.1)	2 (2.7)	1 (1.4)	
	Israel	9 (6.2)	7 (9.6)	2 (2.7)	
	Italy	3 (2.1)	3 (4.1)	0 (0.0)	
	Latvia	1 (0.7)	1 (1.4)	0 (0.0)	
	Netherlands	3 (2.1)	2 (2.7)	1 (1.4)	
	Poland	9 (6.2)	5 (6.8)	4 (5.5)	
	Portugal	2 (1.4)	1 (1.4)	1 (1.4)	
	Romania	3 (2.1)	3 (4.1)	0 (0.0)	
	Slovakia	1 (0.7)	1 (1.4)	0 (0.0)	
	Slovenia	3 (2.1)	0 (0.0)	3 (4.1)	
	Spain	9 (6.2)	5 (6.8)	4 (5.5)	
	Sweden	4 (2.7)	2 (2.7)	2 (2.7)	
	Switzerland	5 (3.4)	2 (2.7)	3 (4.1)	
**Gender (%)**	Male	109 (74.7)	57 (78.1)	52 (71.2)	n.s.
	Female	37 (25.3)	16 (21.9)	21 (28.8)	
**Age of death (mean (SD))**		68.05 (16.41)	68.92 (16.52)	67.18 (16.36)	n.s.
**Next-of-kin/Relationship to the deceased (%)**	Husband/wife/partner	59 (40.4)	32 (43.8)	27 (37.0)	n.s.
	Son/Daughter	28 (19.2)	14 (19.2)	14 (19.2)	
	Son-/Daughter-in-law	2 (1.4)	2 (2.7)	0 (0.0)	
	Son/Daughter of husband, wife or partner	2 (1.4)	1 (1.4)	1 (1.4)	
	Grandchild	1 (0.7)	1 (1.4)	0 (0.0)	
	Sibling	6 (4.1)	3 (4.1)	3 (4.1)	
	Other relative	11 (7.5)	6 (8.2)	5 (6.8)	
	Other non-relative	37 (25.3)	14 (19.2)	23 (31.5)	
**Frequency of Contact in the last year (%)**	Never or Refused to disclose	8 (5.5)	2 (2.7)	6 (8.2)	n.s.
	Less than once a month	7 (4.8)	5 (6.8)	2 (2.7)	
	About once a month	4 (2.7)	0 (0.0)	4 (5.5)	
	About every two weeks	9 (6.2)	3 (4.1)	6 (8.2)	
	About once a week	6 (4.1)	1 (1.4)	5 (6.8)	
	Several times a week	25 (17.1)	9 (12.3)	16 (21.9)	
	Daily	87 (59.6)	53 (72.6)	34 (46.6)	
**How long Ill before death (%)**	Was not ill before death	7 (4.8)	3 (4.1)	4 (5.5)	<0.001
	Less than one month	87 (59.6)	60 (82.2)	27 (37.0)	
	One month or more, but less than 6 months	12 (8.2)	2 (2.7)	10 (13.7)	
	Six months or more, but less than a year	5 (3.4)	0 (0.0)	5 (6.8)	
	One year or more, Don’t know, or Refused	35 (24.0)	8 (11.0)	27 (37.0)	
**Time in Hospital last year (%)**	Less than one week	8 (5.5)	2 (2.7)	6 (8.2)	n.s.
	From one week to one month, Don’t Know	132 (90.4)	67 (91.8)	65 (89.0)	
	From one month to three months	4 (2.7)	2 (2.7)	2 (2.7)	
	From three months to a full year	2 (1.4)	2 (2.7)	0 (0.0)	
**Care from GP in the last year (%)**	Don’t know	8 (5.5)	4 (5.5)	4 (5.5)	n.s.
	Yes	93 (63.7)	48 (65.8)	45 (61.6)	
	No	45 (30.8)	21 (28.8)	24 (32.9)	
**Hospital stays for therapy in the last year (%)**	Yes	131 (89.7)	61 (83.6)	70 (95.9)	n.s.
	No	15 (10.3)	12 (16.4)	3 (4.1)	
**Took Medications in the last year (%)**	Don’t know	10 (6.8)	2 (2.7)	8 (11.0)	n.s.
	Yes	99 (67.8)	52 (71.2)	47 (64.4)	
	No	37 (25.3)	19 (26.0)	18 (24.7)	
**Difficulties in ADL (%)**	Refusal	2 (1.4)	1 (1.4)	1 (1.4)	n.s.
	Don’t know	7 (4.8)	3 (4.1)	4 (5.5)	
	No	37 (25.3)	20 (27.4)	17 (23.3)	
	Yes	100 (68.5)	49 (67.1)	51 (69.9)	
**Hours of Help/Day needed (mean (SD))**		6.23 (5.70)	6.96 (6.41)	5.49 (4.83)	n.s.
**Decedent Had a Will (%)**	Refusal	1 (0.7)	1 (1.4)	0 (0.0)	n.s.
	Don’t know	9 (6.2)	1 (1.4)	8 (11.0)	
	Yes	20 (13.7)	10 (13.7)	10 (13.7)	
	No	116 (79.5)	61 (83.6)	55 (75.3)	
**Owned.Home (%)**	Don’t know	1 (0.7)	1 (1.4)	0 (0.0)	n.s.
	Yes	95 (65.1)	52 (71.2)	43 (58.9)	
	No	50 (34.2)	20 (27.4)	30 (41.1)	
**# Children Still Alive at death of participant** **(mean (SD))**		1.90 (1.92)	2.05 (1.96)	1.75 (1.89)	n.s.
**EURO-Depression Scale Score**		2.15 (1.97)	2.12 (2.02)	2.17 (1.93)	n.s.
**Suicidal Ideation (%)**	Data not reported	92 (63.0)	48 (65.8)	44 (60.3)	n.s.
	Reported some degree of wish to die	6 (4.1)	2 (2.7)	4 (5.5)	
	Denied	48 (32.9)	23 (31.5)	25 (34.2)	
**Any Long-term illness (%)**	Yes	122 (83.6)	62 (84.9)	60 (82.2)	n.s.
	No	24 (16.4)	11 (15.1)	13 (17.8)	
**Last Wave (%)**	#2, year 2008	9 (6.2)	6 (8.2)	3 (4.1)	n.s.
	#3, year 2010	18 (12.3)	9 (12.3)	9 (12.3)	
	#5, year 2015	28 (19.2)	11 (15.1)	17 (23.3)	
	#6, year 2016	33 (22.6)	18 (24.7)	15 (20.5)	
	#7, year 2017	26 (17.8)	12 (16.4)	14 (19.2)	
	#8, year 2018	29 (19.9)	15 (20.5)	14 (19.2)	
	#9, year 2020	3 (2.1)	2 (2.7)	1 (1.4)	

ADL, Activities of Daily; GP, General Practitioner Living; n.s., not significant; SD, standard deviation. Statistical significance was assessed with a Student’s t-test for continuous variables or a Chi-square test for frequency data.

Regarding the duration of the illness before death, the most frequent answers were one year or more (24%) and less than one month (59.6%). Regarding the total time spent hospitalized, 90.4% of the sample stayed in hospital between 1 and 4 weeks in the last year. Sensitivity analysis for groups of illnesses and reasons for hospital stays could not be conducted because of the reduced sample size and/or unavailability of further details. More than half of the sample were visited by their GP at least once in the year before death, and approximately 68% took medication for any physical or mental illness. Difficulties in activities of daily living (ADL) were present in 68.5% of the sample. Regarding long-term diseases, 83.6% of the sample reported having at least one. Only 6 participants (4.1%) reported making any mention of suicidal feelings or the wish to be dead. In contrast, 32.9% of the sample denied such feelings. For 63% of the sample, the interviewer did not report if suicidal ideation was present/absent or if the participant did not know or refused to answer.

Regarding the time gap between the last in-person interview with the participant and the end-of-life interview with the next-of-kin (due to the death of the participant), only one year passed for most death occurrences (43 deaths by suicide and 45 accidents). Two years passed for 11 deaths by accident and ten deaths by suicide (next-of-kin interviews that took place at waves 3 and 9); three years for 17 deaths by accident and 20 by suicide (next-of-kin interviews that took place at waves 2 and 5). Furthermore, 71.2% of the participants did not have a will at the time of death.

No significant differences emerged between participants who died by suicide and those who died by accident, except for the length of illness before death: participants who died by suicide (37%) had been ill for more than one year, while these figures were significantly lower for people who died by accident (11%), who, on the other hand, had a more recent illness onset (82.2% in the previous month compared to 37% of those who died by suicide). Before implementing the random forest model, we conducted a univariate analysis to discern which factors differed between the two population samples before the one-on-one matching (**Supplementary**-**Table S2**) and after the matching (**Table 1**). Before matching, people who died by suicide (n=73) differed significantly from people who died in accidents (n=420) in age at death (69.09 ± 11.78 and 76.72 ± 11.72 respectively – t = 5.041, df = 95.563, p-value <.001) and duration of illness before death (longer duration of illness for people who died by suicide – X-squared = 89.74, df = 5, p <.001). After one-to-one-matching, which included matching also based on age at death, only the duration of the illness before death was still significantly different (X-squared = 33.308, df = 4, p <.001). In the univariate analysis, no other statistically significant differences between the two samples emerged before or after one-on-one matching in the variables of interest.

### Random forest model

We implemented a random forest algorithm to determine which factors reported at wave *n-1* (henceforth “baseline”) or retrospective information collected from the next-of-kin during the end-of-life interview could be leveraged to improve the prediction of death from suicide at wave *n*.

**Figure 1:**
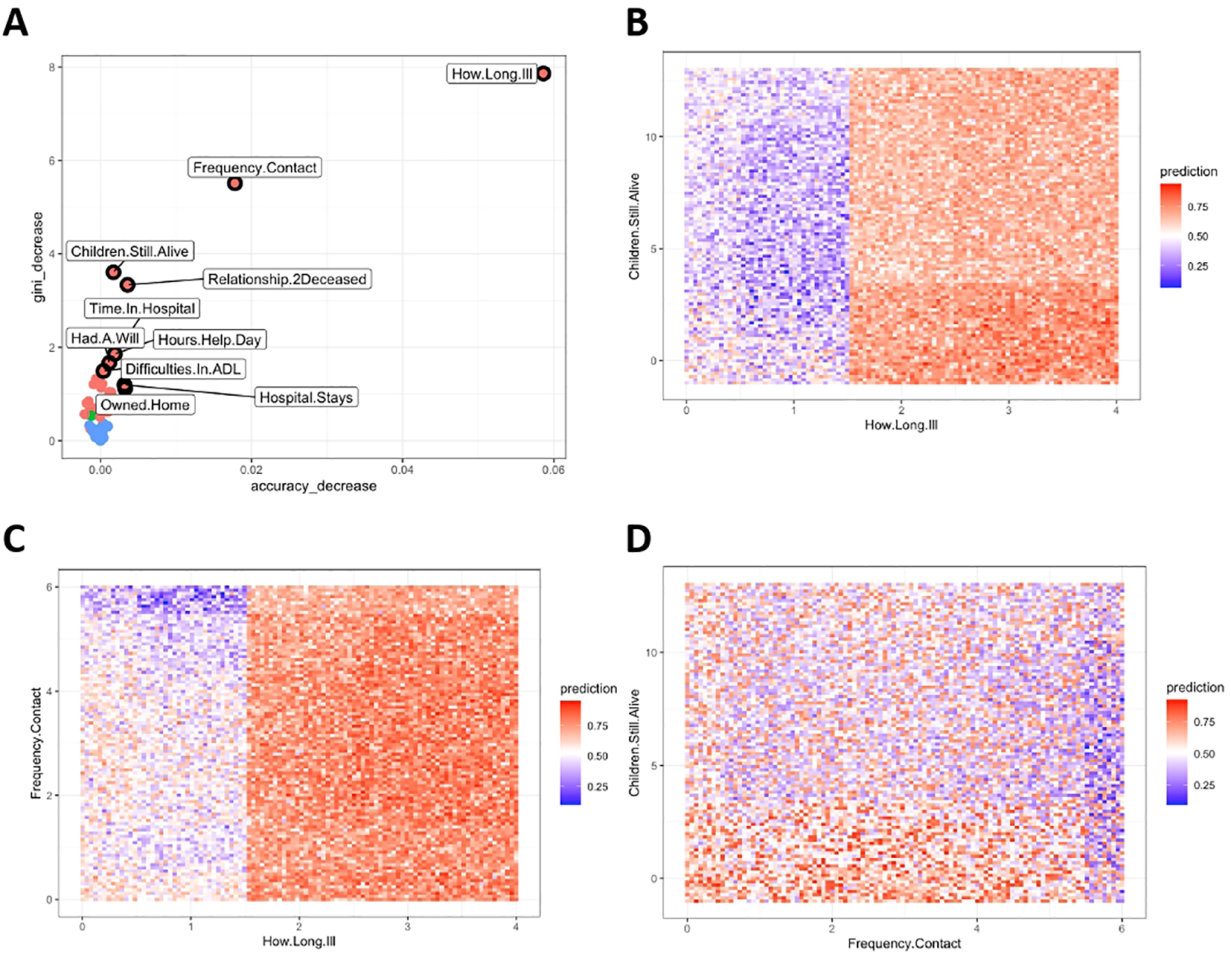
Predictors distinguishing death from suicide from death from accident. ADL, Activities of Daily Living. Gini Importance = This index estimates how much a random forest relies on a particular feature in classification. In particular, a decrease of Gini Importance measures how much a variable helps in the correct classification of cases (by assessing the loss of purity if that variable is excluded from the analysis). It measures the average gain of purity by splits of a given variable. If the variable (e.g., duration of illness) is useful, it splits mixed labeled nodes (mixed group of participants that died by suicide or accident) into pure single class nodes (two groups, one with people who died by suicide and one with people who died by accident). Accuracy decrease = It indicates the loss of model performance without each variable (excluded from the analysis one at the time). **(A)** The plot displays variables according to their contribution to the model’s performance, as determined by metrics such as accuracy decrease and Gini decrease. Variables closer to the upper right corner have a greater impact to model performance, indicating their higher importance in distinguishing between outcomes (e.g., deaths by suicide vs. accidents). For instance, removing “Duration of Illness – How.Long.Ill” decreases the model’s accuracy by 0.06 (6%) and its Gini index by 8 (in range 0-100). **(B)** This panel illustrates the predicted probability of death by suicide, influenced by the duration of illness and the number of children still alive. The color gradient from 0 (deep blue) to 1 (red) represents increasing suicide risk. For example, the lower right corner, where the probability approaches 1, indicates a high likelihood of classifying a death by suicide if the duration of illness is long and the number of children is low. The illness duration is categorized as follows: 1 = less than one month, 2 = 1-6 months, 3 = more than six months, 4 = a year or more. A longer duration typically increases risk, but this can be mitigated if the individual has three or more children alive. **(C)** This plot shows how suicide risk varies with the duration of illness and the frequency of contact with the next-of-kin. The frequency ranges from 0 (=never) to 6 (=daily), with 2 representing about once a month and 4 about once a week. Higher frequencies of contact, especially daily, generally indicated a reduced risk of suicide. However, this protective effect diminishes with longer illness durations. **(D)** This panel examines the relationship between contact frequency with next-of-kin and the number of children still alive on suicide risk. No distinct patterns are evident, but the data suggest that lower contact frequency and fewer children are associated with increased suicide risk.

The algorithm was trained on 80% of the sample to predict two possible outcomes: death from suicide and death from accident. Using the split mentioned above, the algorithm was asked to correctly categorize 58 deaths by accidents and 59 deaths by suicide. At the training level, the overall classification error (out-of-bag estimate of error - OOB) was 33.33% (s.d. = 3.3%), with no striking difference between the misclassification of death from accidents or death from suicide: 39 of 59 deaths by suicide were correctly categorized (error rate 33.89%); out of 58 deaths by accidents, 39 were correctly identified as such (error rate 32.75%). The most important variables (as measured via accuracy and Gini decrease) to tell apart deaths by accident and death from suicide were the following (**Figure 1A**): the relationship between the next-of-kin and the decedent (the next-of-kin of people who died by suicide was more likely to be a non-relative), the total length of hospitalization in the previous year, and if any access to the hospital was necessary for therapy administration (the need for hospitalization increased the risk of suicide, especially shorter-length hospitalizations), difficulties in the activities of daily living, how many hours of help the decedent needed (the fewer hours of help needed, the higher the risk of suicide) if they owned a home and had a will. The three most important variables identified by the algorithm were: longer duration of illness, less than daily contact with the next-of-kin, and less than three children still alive, which were all independent predictors of suicide. Moreover, we found relevant interactions between the duration of the illness, the number of children still alive, and the frequency of contact with the next of kin (**Figure 1B**). In particular, a longer-than-one-month illness duration increases the risk of suicide with respect to a shorter length; this risk could be further exacerbated if the participant had fewer than three children still alive (on the other hand, having three or more children mitigated the probability of suicide regardless of the longer-than-one-month illness duration). A similar interaction was present throughout the duration of the illness and contact with the next-of-kin (**Figure 1C**). Noteworthily, less than daily contact with the next-of-kin increased the risk of suicide. Lastly, we found an interaction between the frequency of contact with the next-of-kin and the number of children still alive (**Figure 1D**). The algorithm identified an increased risk of suicide with a reduced frequency of contact and a smaller number of children still alive.

By leveraging the above variables, the random forest algorithm was required to categorize the deaths of the remaining 20% of the sample. Eleven of the 15 deaths by accidents were correctly identified. Regarding deaths from suicide, the prediction was corrected for 12 out of 14 deaths. This classification yielded an overall accuracy of 0.79 (CI [0.60 – 0.92], p = 0.002) with a sensitivity of.80 and a specificity of.78.

## Discussion

In this study, we queried the *Survey of Health, Ageing, and Retirement in Europe* (SHARE) dataset to reveal the predictors of death from suicide in late adulthood. We applied a supervised machine learning (random forest) algorithm to automatically extract the variables deemed important in discerning death from suicide from death from accident after appropriate population matching according to sex, age of death, number of physical illnesses, and suicidal ideation. Our analyses returned several physical and social health variables that are central in distinguishing accidental death from suicide. These variables included the duration of illness, the frequency of contact with the next-of-kin, the number of children, the time spent in the hospital in the last year, the hours of help per day needed, the presence of difficulties in the activities of daily living, the relationship between the next-of-kin and the decedent. We showed that great importance is placed on the duration of the illness (**Figure 1**): illnesses lasting more than a month in the year before death were more likely to predict death from suicide than death from accident, and longer-term illnesses (i.e., > one year) posit a higher risk of suicide. Nevertheless, the data from this sample seem to indicate that death from suicide was more probable with a duration of illness of 1-6 months. We argue that this should be considered at least in light of the age of the deceased and the nature of the illness itself: rapid-onset illnesses that hinder the daily activities of individuals or require extensive medical care with no apparent prospect of recovery (50), challenge daily living, and could further exacerbate a deterioration in physical or mental conditions of older people. Contact with healthcare providers has already been shown to be more likely to occur less than a month before death from suicide in older adults than in younger adults (51), probably correlated with a greater need for care. Although detailed analyses would be essential to determine the impact of specific illnesses on suicide, this was unfeasible, as it would have required a larger sample size. However, by leveraging the SHARE dataset, other authors reported that specific system diseases are more likely to be associated with suicidal ideation (52), although no correlation between suicidal ideation and death from suicide could be drawn (53). The most important factors were the frequency of contact with the next-of-kin (who also completed an end-of-life interview by proxy), their relationship with the decedent, and the number of children the individual had before death. In this sample, the next-of-kin of the individuals who died by suicide was a non-relative 31.5% of the time, with respect to 19.2% for those who died by accident, indicating a lesser presence of family members in the life of those who died by suicide. All these variables are related to the participant’s social connections before death and indicate the role of interactions with family members (41). The role that family might play in suicide prevention has previously been evidenced, specifically by reducing feelings of loneliness, increasing belongingness, and possibly reducing anxiety and depressive symptoms (16). Here, we report that there could be a “dose threshold” for the frequency of contact with next-of-kin, above which the probability of death from suicide could be diminished. Specifically, we showed that people who had daily or multiple contacts a week with their next-of-kin had a reduced likelihood of dying by suicide, also considering the duration of the disease and the number of children still alive. The algorithm also identified that participants who had no children alive at their death were more likely to have died by suicide than those with children, particularly those with three or more. To further corroborate the hypothesis of the pivotal role of family support in suicide prevention in late life, the algorithm identified the relationship between the next-of-kin and the decedent as a predictor in differentiating death from suicide from death from accident. Few other studies investigated the link between loneliness and death from suicide (54), and a recent meta-analysis also highlighted that no studies published up to 2020 included suicide death as a distinct outcome measure (10). However, most of the published literature indicates a moderate to strong association between loneliness and non-lethal suicidal behaviors. These findings presented herein evidenced that factors related to loneliness may be a predictor of death from suicide, at least in older adults, as postulated by recent theories of suicide (55, 56). Further studies are needed to corroborate our findings in other geographical areas.

### Limitations

Although this analysis is based on a prospective, harmonized dataset, it is worth noting that this data survey was not conceptualized for studying suicidal behaviors. Therefore, some important variables related to this phenomenon were not collected. For example, we highlight that no measures for grief, which was shown to be an important factor for suicide death (41, 57), were available; in addition, there was no direct information on previous or current mental disorders among the participants, but only data on current mood could be drawn from the EURO-D scale. Moreover, suicidal ideation was not thoroughly assessed with specific questions aimed at knowing if the respondent had the intention or plan to end their life, or, on the other hand, the frequency and intrusiveness of those thoughts. In particular, we highlight that for more than half of the sample, data on suicide ideation is either missing or the participant did not know if they had suicidal thoughts/refused to answer. This could be interpreted in light of the difficulty that elderly people experience in disclosing the wish to die. However, no strong conclusions can be made regarding the reason for missing data at this point. Similarly, details regarding the circumstances and dynamics of the accidents that resulted in the death of a person were not available. Therefore, it cannot be certainly excluded that some deaths by accidents were not suicide attempts. However, such events tend to be single-car accidents (less than 3% of all road fatalities are thought to be suicides (58)). In this sample, only three people (4.1%) who died by accident died outside of their house/hospital (compared to 14 people (19.2%) who died by suicide), making the chances of misclassification negligible. Furthermore, end-of-life interviews were conducted with the help of the next-of-kin and, obviously, in a retrospective manner. This likely implied recall bias. In addition, respondents might have reported purposefully inflated/deflated figures regarding the decedent’s care to deal with the interviewers’ desirability/sense of righteousness, although it would be impossible to prove if this has ever occurred.

Second, death from suicide represents approximately 1% of all deaths. Given the rarity of the event, thousands of deaths had to be recorded to have enough data to draw decently solid conclusions. In this dataset, 74 suicides were registered out of 16,548 deaths. Although this sample size might prove sufficient to evince strong associations and contributions of socio-demographic and clinical factors to suicide, some nuances might not be evidenced: for example, it was unfeasible to conduct further analysis on the role of the duration of illnesses of specific systems (e.g., cardiovascular, respiratory, etc.) on suicide. Moreover, some data were not reported for all participants and had to be imputed. This is a typical case for large datasets.

Lastly, it should be noted that the statistical approach presented herein differentiates deaths by suicide from deaths by accident based on the quality and quantity of data provided, regardless of previous findings or authors’ views.

## Conclusions

We employed a machine learning algorithm to demonstrate the predictors of late-life suicide in a large longitudinal European cohort. When tested, the random forest algorithm yielded an overall accuracy of 0.79 (CI [0.60 – 0.92], p = 0.002). It highlighted that the most important variables used to discern deaths by suicide from deaths by accident were social connectedness-related (frequency of contact with the next-of-kin, the relationship with the next-of-kin, the number of children still alive) and physical illness-related (duration of illness before deaths, length of hospitalization in the 12 months preceding deaths, difficulties in the activities of daily living). The findings presented here provide a hierarchical importance of predictors for late-life suicide and highlight social connection and physical health as critical variables for assessing suicide risk. Replication of these findings and deepening our understanding of these predictors through experts by experience (i.e., survivors of near-lethal suicide attempts) will be instrumental in designing accurate prediction models and tailored interventions for suicide prevention.

## Data Availability

Publicly available datasets were analyzed in this study. This data can be found here: https://share-eric.eu/data/data-access.
