# Supplementary material for: Late-life suicide: machine learning predictors from a large European longitudinal cohort
Source: Front Psychiatry. 2024 Sep 17;15:1455247. doi: 10.3389/fpsyt.2024.1455247 (PMC11442232; doi:10.3389/fpsyt.2024.1455247)
Supplement: Supplementary file 1 [file DataSheet1.docx]

**Supplementary material for:**

**Late-life suicide: machine learning predictors from a large European longitudinal cohort**

**Methods S1. Random forest algorithms**

Random Forest is a widely used machine learning algorithm that aggregates the outputs of multiple decision trees to derive a single prediction.

Decision trees sit at the foundation of random forests. A decision tree begins with a root question, such as "Should I submit a paper to this journal?" From this starting point, a series of subsequent questions, like "Is the time to first decision long?" or "Is the review process going to improve the manuscript?" guide the decision-making process. Each question represents a node, which splits the data based on the answers, eventually leading to a conclusion or leaf node. Decision trees are usually built using the Classification and Regression Tree (CART) algorithm, which seeks the optimal splits to categorize the data. Metrics such as Gini impurity, information gain, or mean square error (MSE) assess the effectiveness of these splits.

When multiple decision trees are combined into an ensemble, such as in the Random Forest algorithm, a collective prediction is generated.

The aggregate/collective predictions — in our use-case through majority voting — usually yield more accurate results by reducing variance and mitigating the effects of noise in the dataset.

To put it together, the random forest algorithm creates a collection of decision trees with low intercorrelation. Building a random forest involves selecting a random subset of features for each decision tree, which ensures diversity among the trees. This approach contrasts with individual decision trees, which consider all potential feature splits.

By including different subsets of features across multiple trees, Random Forest reduces the risks of overfitting and bias, leading to more robust and precise predictions (see also <https://www.ibm.com/topics/random-forest>).

For example, random Forests can classify patients based on symptoms and test results, aiding in the diagnosis of cancer subtypes (1) or in the data analysis of next-generation sequencing (2). The accuracy of the algorithm varies according to the quality and quantity of the collected data on which it is trained.

Random forest performance can be tested through sensitivity and specificity (along with positive/negative predictive value and overall accuracy), similar to the comparison of different procedures on an outcome of interest. Specifically, the prediction of the random forest model (i.e., if one person died by suicide or accident) is compared to the real-world outcome (if that person actually died in that manner). By doing so for each participant, the value of true positives (the number of people correctly identified by the model as having died in a specific manner) and false positives (i.e., misclassifications of the model) is derived (and vice versa for true/false negatives). Overall accuracy is determined as the proportion of true results (both true positives and true negatives) among the total number of cases examined. Sensitivity measures the proportion of true positives correctly identified by the model. Specificity measures the proportion of true negatives correctly identified by the model.

**Table S1. Variables shared across waves and considered in the analysis**

| **End-of-life Interview Variables** | | mh014_: | Concentration on entertainment |
| --- | --- | --- | --- |
| xt002_: | Relationship to the deceased | mh015_: | Concentration on reading |
| xt005_: | Frequency of contact between next-fo-kin and decedent | mh016_: | Enjoyment |
| xt013_: | Duration of illness | mh017_: | Tearfulness |
| xt016_: | Time spent in hospital in the last 12 months | ph003_: | Health in general question 2 |
| xt018_1: | Type of medical care in the last 12 months: care from a general practitioner | ph004_: | Long-term illness |
| xt018_2: | Type of medical care in the last 12 months: care from specialist physicians | ph005_: | Limited in activities because of health |
| xt018_3: | Type of medical care in the last 12 months: hospital stays | ph006d1: | Heart attack: ever diagnosed |
| xt018_4: | Type of medical care in the last 12 months: care in a nursing home | ph006d2: | High blood pressure or hypertension: ever diagnosed |
| xt018_5: | Type of medical care in the last 12 months: hospice stays | ph006d3: | High blood cholesterol: ever diagnosed |
| xt018_6: | Type of medical care in the last 12 months: medication | ph006d4: | Stroke: ever diagnosed |
| xt018_7: | Type of medical care in the last 12 months: aids and appliances | ph006d5: | Diabetes or high blood sugar: ever diagnosed |
| xt018_8: | Type of medical care in the last 12 months: home care or help due to disability | ph006d6: | Chronic lung disease: ever diagnosed |
| xt020dno: | Difficulties doing activities: none of these | ph006d10: | Cancer: ever diagnosed |
| xt022_: | Anyone helped with ADL | ph006d11: | Stomach or duodenal ulcer, peptic ulcer: ever diagnosed |
| xt23d3: | Who has helped with ADL: mother/father of the deceased | ph006d13: | Cataracts: ever diagnosed |
| xt023d11: | Who has helped with ADL: brother of deceased | ph006d14: | Hip fracture or femoral fracture: ever diagnosed |
| xt024_: | Time the deceased received help | ph006dno: | None: ever diagnosed |
| xt025_: | Hours of help necessary during a typical day | ph006dot: | Other: ever diagnosed |
| xt026_: | The deceased had a will | ph008d18: | Cancer in: colon or rectum |
| xt027d1: | Beneficiaries of the estate: yourself (proxy) | ph009_1: | Age heart attack or other heart problems |
| xt027d2: | Beneficiaries of the estate: spouse/partner of the deceased | ph009_2: | Age high blood pressure |
| xt027d3: | Beneficiaries of the estate: children of the deceased | ph009_3: | Age high blood cholesterol |
| xt027d4: | Beneficiaries of the estate: grandchildren of the deceased | ph009_6: | Age chronic lung disease |
| xt027d5: | Beneficiaries of the estate: siblings of the deceased | ph009_13: | Age cataracts |
| xt027d6: | Beneficiaries of the estate: other relatives of the deceased | ph011d1: | Drugs for: high blood cholesterol |
| xt027d7: | Beneficiaries of the estate: other non-relatives | ph011d2: | Drugs for: high blood pressure |
| xt027d8: | Beneficiaries of the estate: church, foundation or charitable organization | ph011d3: | Drugs for: coronary diseases |
| xt027d9: | Beneficiaries of the estate: deceased did not leave anything at all (SPONTANEOUS | ph011d4: | Drugs for: other heart diseases |
| xt030_: | The deceased owned home | ph011d6: | Drugs for: diabetes |
| xt033_: | Deceased owned any life insurance policies | ph011d7: | Drugs for: joint pain |
| xt039_: | Number of children (still alive) deceased had at the end | ph011d8: | Drugs for: other pain |
| xt041_: | Funeral was accompanied by a religious ceremony | ph011d9: | Drugs for: sleep problems |
| **Behavioral Risks Variables (cont. third column)** | | ph011d10: | Drugs for: anxiety or depression |
| br001_: | Ever smoked daily | ph011d11: | Drugs for: osteoporosis (hormonal) |
| br002_: | Smoke at the present time | ph011d13: | Drugs for: stomach burns |
| br003_: | How many years smoked | ph011d14: | Drugs for: chronic bronchitis |
| br015_: | Sports or activities that are vigorous | ph011dno: | Drugs for: none |
| br016_: | Activities requiring a moderate level of energy | ph011dot: | Drugs for: other |
| br017_: | Who responded to the questions above | ph041_: | Wears glasses/contact lenses |
| **Cognitive functioning variables** | | ph043_: | Eyesight distance |
| cf001_: | Self-rated reading skills | ph044_: | Eyesight reading |
| cf002_: | Self-rated writing skills | ph045_: | Use hearing aid |
| cf003_: | Date: day of month | ph046_: | Hearing |
| cf004_: | Date: month | ph048d1: | Difficulties: walking 100 metres |
| cf005_: | Date: year | ph048d2: | Difficulties: sitting two hours |
| cf006_: | Date: day of the week | ph048d3: | Difficulties: getting up from chair |
| cf010_: | Verbal fluency score: number of animals | ph048d4: | Difficulties: climbing several flights of stairs |
| cf012_: | Numeracy: chance disease 10% of 1000 | ph048d5: | Difficulties: climbing one flight of stairs |
| cf013_: | Numeracy: half price | ph048d6: | Difficulties: stooping, kneeling, crouching |
| cf014_: | Numeracy: 6000 is two-thirds what is total price | ph048d7: | Difficulties: reaching or extending arms above shoulder |
| cf015_: | Numeracy: amount in the savings account | ph048d8: | Difficulties: pulling or pushing large objects |
| cf017_: | Contextual factors during the cognitive function test | ph048d9: | Difficulties: lifting or carrying weights over 5 kilos |
| cf018d1: | Who present during cf: respondent alone | ph048d10: | Difficulties: picking up a small coin from a table |
| cf018d2: | Who present during cf: partner present | ph048dno: | Difficulties: none of these |
| cf018d3: | Who present during cf: child(ren) present | ph049d1*: | Difficulties: dressing, including shoes and socks |
| cf018d4: | Who present during cf: other(s) | ph049d2*: | Difficulties: walking across a room |
| **Household variables** | | ph049d3*: | Difficulties: bathing or showering |
| partnerinhh: | Partner in household | ph049d4*: | Difficulties: eating, cutting up food |
| relrpers: | Relation to coverscreen respondent | ph049d5*: | Difficulties: getting in or out of bed |
| hhsize: | Household size | ph049d6*: | Difficulties: using the toilet, incl getting up or down |
| fam_resp: | Family respondent | ph049d7: | Difficulties: using a map in a strange place |
| fin_resp: | Financial respondent | ph049d8: | Difficulties: preparing a hot meal |
| hou_resp: | Household respondent | ph049d9: | Difficulties: shopping for groceries |
| interview: | Interview done in wave 1 | ph049d10: | Difficulties: telephone calls |
| ft002_: | Given financial gift 250 or more | ph049d11: | Difficulties: taking medications |
| ft015_: | Ever received gift or inheritance (worth 5000 euros or more) | ph049d12: | Difficulties: doing work around the house or garden |
| ft016_1: | Year, inheritance or large gift 1 received | ph049d13: | Difficulties: managing money |
| ft021_: | Who answered the questions in ft | ph049dno: | Difficulties: none of these |
| **Physical and Mental Health variables** | | ph054_: | Who answered the questions in ph |
| gs002_: | Record respondent status | sp002_: | Received help from others (outside hh) |
| gs004_: | Dominant hand | sp005_1: | How often received help: from person 1 |
| gs006_: | 1st measurement: left hand | sp005_2: | How often received help: from person 2 |
| gs007_: | 2nd measurement: left hand | sp005_3: | How often received help: from person 3 |
| gs008_: | 1st measurement: right hand | sp007_1: | Any other helper from outside the household |
| gs009_: | 2nd measurement: right hand | sp007_2: | Any other helper from outside the household |
| hc012_: | Stayed overnight in the hospital last 12 months | sp008_: | Given help last twelve months |
| hc013_: | Times being a patient in the hospital | sp011_1: | How often given help to person 1 |
| hc014_: | Total nights stayed in the hospital | sp011_2: | How often given help to person 2 |
| hc029_: | In a nursing home during the last 12 months | sp013_1: | Have you given help to others |
| hc031_: | Weeks stayed in a nursing home | sp014_: | Looked after grandchildren |
| mh002_: | Sad or depressed last month | sp015d1: | Looked after child of child 1 |
| mh003_: | Hopes for the future | sp015d2: | Looked after child of child 2 |
| mh004_: | Suicidal feelings | sp015d3: | Looked after child of child 3 |
| mh005_: | Feels guilty | sp015d4: | Looked after child of child 4 |
| mh006_: | Blame for what | sp016_2: | How often did you look after child of child 2 |
| mh007_: | Trouble sleeping | sp016_4: | How often did you look after child of child 4 |
| mh008_: | Less or same interest in things | sp018_: | Given help to someone with personal care in the household |
| mh010_: | Irritability | sp020_: | Someone in this household helped you regularly with personal care |
| mh011_: | Appetite | sp022_: | Who answered the questions in sp |
| mh013_: | Fatigue | **Outcome**: | Death by Suicide/Accident |

*Please note that ph049d[X] questions represent the variables encoding difficulties in activities of daily living.

**Table S2. Sample characteristics before one-on-one matching**

|  |  | **Accidents** | **Suicides** | ***p-value*** |
| --- | --- | --- | --- | --- |
| **Sample N** |  | 420 | 73 |  |
| **Country (%)** | Austria | 22 (5.2) | 7 (9.6) | n.s. |
|  | Germany | 14 (3.3) | 1 (1.4) |  |
|  | Sweden | 11 (2.6) | 2 (2.7) |  |
|  | Netherlands | 10 (2.4) | 1 (1.4) |  |
|  | Spain | 34 (8.1) | 4 (5.5) |  |
|  | Italy | 20 (4.8) | 0 (0.0) |  |
|  | France | 23 (5.5) | 9 (12.3) |  |
|  | Denmark | 20 (4.8) | 4 (5.5) |  |
|  | Greece | 41 (9.8) | 0 (0.0) |  |
|  | Switzerland | 11 (2.6) | 3 (4.1) |  |
|  | Belgium | 26 (6.2) | 11 (15.1) |  |
|  | Israel | 17 (4.0) | 2 (2.7) |  |
|  | Czech Republic | 36 (8.6) | 7 (9.6) |  |
|  | Poland | 17 (4.0) | 4 (5.5) |  |
|  | Ireland | 1 (0.2) | 0 (0.0) |  |
|  | Hungary | 14 (3.3) | 1 (1.4) |  |
|  | Portugal | 7 (1.7) | 1 (1.4) |  |
|  | Slovenia | 18 (4.3) | 3 (4.1) |  |
|  | Estonia | 47 (11.2) | 13 (17.8) |  |
|  | Croatia | 2 (0.5) | 0 (0.0) |  |
|  | Lithuania | 4 (1.0) | 0 (0.0) |  |
|  | Bulgaria | 4 (1.0) | 0 (0.0) |  |
|  | Cyprus | 1 (0.2) | 0 (0.0) |  |
|  | Finland | 1 (0.2) | 0 (0.0) |  |
|  | Latvia | 2 (0.5) | 0 (0.0) |  |
|  | Malta | 1 (0.2) | 0 (0.0) |  |
|  | Romania | 13 (3.1) | 0 (0.0) |  |
|  | Slovakia | 3 (0.7) | 0 (0.0) |  |
| **Gender (%)** | Male | 231 (55.0) | 52 (71.2) | n.s. |
|  | Female | 189 (45.0) | 21 (28.8) |  |
| **Age of death (mean (SD))** |  | 76.72 (11.72) | 69.09 (11.78) | <0.001 |
| **Next-of-kin/Relationship to the deceased (%)** | Husband/wife/partner | 136 (32.4) | 27 (37.0) | n.s. |
|  | Son/Daughter | 128 (30.5) | 14 (19.2) |  |
|  | Son-/Daughter-in-law | 9 (2.1) | 0 (0.0) |  |
|  | Son/Daughter of husband, wife or partner | 5 (1.2) | 1 (1.4) |  |
|  | Grandchild | 10 (2.4) | 0 (0.0) |  |
|  | Sibling | 13 (3.1) | 3 (4.1) |  |
|  | Other relative | 30 (7.1) | 5 (6.8) |  |
|  | Other non-relative | 89 (21.2) | 23 (31.5) |  |
| **Frequency of Contact in the last year (%)** | Never or Refused to disclose | 7 (1.7) | 6 (8.2) | n.s. |
|  | Less than once a month | 21 (5.0) | 2 (2.7) |  |
|  | About once a month | 9 (2.1) | 4 (5.5) |  |
|  | About every two weeks | 15 (3.6) | 6 (8.2) |  |
|  | About once a week | 22 (5.3) | 5 (6.8) |  |
|  | Several times a week | 79 (18.9) | 16 (21.9) |  |
|  | Daily | 266 (63.5) | 34 (46.6) |  |
| **How long Ill before death (%)** | Was not ill before death | 19 (4.5) | 4 (5.5) | <0.001 |
|  | Less than one month | 347 (82.6) | 27 (37.0) |  |
|  | One month or more but less than 6 months | 21 (5.0) | 10 (13.7) |  |
|  | Six months or more but less than a year | 8 (1.9) | 5 (6.8) |  |
|  | One year or more, Don’t know or Refused | 25 (6.0) | 27 (37.0) |  |
| **Time in Hospital last year (%)** | Less than one week | 8 (1.9) | 6 (8.2) | n.s. |
|  | From one week to one month, Don’t Know | 388 (92.4) | 65 (89.0) |  |
|  | From one  month to three months | 13 (3.1) | 2 (2.7) |  |
|  | From three months to a full year | 11 (2.6) | 0 (0.0) |  |
| **Care from GP in the last year (%)** | Don't know | 13 (3.1) | 4 (5.5) | n.s. |
|  | Yes | 317 (75.5) | 45 (61.6) |  |
|  | No | 90 (21.4) | 24 (32.9) |  |
| **Hospital stays for therapy in the last year (%)** | Yes | 376 (89.5) | 70 (95.9) | n.s. |
|  | No | 44 (10.5) | 3 (4.1) |  |
| **Took Medications in the last year (%)** | Don't know | 11 (2.6) | 8 (11.0) | n.s. |
|  | Yes | 326 (77.6) | 47 (64.4) |  |
|  | No | 83 (19.8) | 18 (24.7) |  |
| **Difficulties in ADL (%)** | Refusal | 2 (0.5) | 1 (1.4) | n.s. |
|  | Don't know | 13 (3.1) | 4 (5.5) |  |
|  | Not selected | 150 (35.7) | 17 (23.3) |  |
|  | Selected | 255 (60.7) | 51 (69.9) |  |
| **Hours of Help/Day needed (mean (SD))** |  | 6.57 (5.94) | 5.49 (4.83) | n.s. |
| **Decedent Had a Will (%)** | Refusal | 2 (0.5) | 0 (0.0) | n.s. |
|  | Don't know | 26 (6.2) | 8 (11.0) |  |
|  | Yes | 67 (16.0) | 10 (13.7) |  |
|  | No | 325 (77.4) | 55 (75.3) |  |
| **# Children Still Alive at death of participant**  **(mean (SD))** |  | 1.92 (1.65) | 1.75 (1.89) | n.s. |
| **Suicidal Ideation (%)** | Data not reported | 266 (63.3) | 44 (60.3) | n.s. |
|  | Reported some degree of wish to die | 27 (6.4) | 4 (5.5) |  |
|  | Denied | 120 (28.6) | 25 (34.2) |  |
|  | Don’t Know | 6 (1.4) | 0 (0.0) |  |
|  | Refused to disclose | 1 (0.2) | 0 (0.0) |  |
| **Any Long-term illness (%)** | Yes | 359 (85.5) | 60 (82.2) | n.s. |
|  | No | 61 (14.5) | 13 (17.8) |  |
| **Last Wave (%)** | #2, year 2008 | 13 (3.1) | 3 (4.1) | n.s. |
|  | #3, year 2010 | 32 (7.6) | 9 (12.3) |  |
|  | #5, year 2015 | 50 (11.9) | 17 (23.3) |  |
|  | #6, year 2016 | 83 (19.8) | 15 (20.5) |  |
|  | #7, year 2017 | 97 (23.1) | 14 (19.2) |  |
|  | #8, year 2018 | 123 (29.3) | 14 (19.2) |  |
|  | #9, year 2020 | 22 (5.2) | 1 (1.4) |  |

ADL = Activities of Daily; GP = General Practicioner Living; n.s. = not significant; SD = standard deviation. Statistical significance was assessed with a Student’s t-test for continuous variables or a Chi-square test for frequency data.

**Supplementary References**

1. Kolisnik T, Sulit AK, Schmeier S, Frizelle F, Purcell R, Smith A, et al. Identifying important microbial and genomic biomarkers for differentiating right- versus left-sided colorectal cancer using random forest models. BMC Cancer. 2023 Jul 11;23(1):647.

2. Pellegrino E, Jacques C, Beaufils N, Nanni I, Carlioz A, Metellus P, et al. Machine learning random forest for predicting oncosomatic variant NGS analysis. Sci Rep. 2021 Nov 8;11(1):21820.
